# Low-dose dasatinib-induced chylothorax, pulmonary hypertension, and pericardial effusion in a patient with chronic myeloid leukemia: A case report and literature review

**DOI:** 10.1097/MD.0000000000041328

**Published:** 2025-01-17

**Authors:** Ting-Wei Pai, Cheng-Wei Huang

**Affiliations:** a Department of Medical Education, Changhua Christian Hospital, Changhua, Taiwan; b Division of Hematology/Oncology, Changhua Christian Hospital, Changhua, Taiwan.

**Keywords:** chronic myeloid leukemia, chylothorax, dasatinib, pericardial effusion, pulmonary hypertension

## Abstract

**Rationale:**

Chylothorax is a rare adverse effect that is associated with dasatinib, a tyrosine kinase inhibitor administered for chronic myeloid leukemia (CML) treatment. Most reported cases have described standard dosing. In this case report, we described a 43-year-old male patient with CML who developed chylothorax after 4 years of low-dose dasatinib therapy. To the best of our understanding, this is the first case to report the simultaneous development of pulmonary hypertension, pericardial effusion, and dasatinib-induced chylothorax.

**Patient concerns:**

A 43-year-old male patient with CML developed chylothorax after 4 years of low-dose dasatinib.

**Diagnoses:**

The patient also developed pulmonary hypertension and pericardial effusion at the same time.

**Interventions:**

Therapeutic interventions included thoracentesis, steroids, diuretics, and sildenafil. Dasatinib was discontinued and replaced with nilotinib, which resolved the chylothorax, pulmonary hypertension, and pericardial effusion.

**Outcomes:**

Chylothorax occurs with long-term and low-dose dasatinib administration. Concurrent pulmonary hypertension and pericardial effusion, although infrequent, may occur. Prompt recognition, dasatinib discontinuation, and therapeutic interventions are crucial for optimizing outcomes.

**Lessons:**

Close monitoring for these rare side effects is paramount even in patients on long-term or low-dose dasatinib.

## 1. Introduction

Chronic myeloid leukemia (CML) is a hematologic malignancy. It is treated primarily with tyrosine kinase inhibitors (TKIs), which target the Breakpoint Cluster Region-Abelson Tyrosine Kinase 1 (BCR::ABL1) fusion protein.^[1]^ Dasatinib, a second-generation TKI, inhibits multiple tyrosine kinases including BCR::ABL1, and is a common treatment choice for CML.^[2]^ Possible adverse effects of dasatinib include pulmonary hypertension^[3]^ and pericardial effusion,^[4]^ although these are relatively uncommon. Chylothorax is also a rare dasatinib-induced side effect, with a currently unclear underlying mechanism.^[5]^ To date, published literature indicates that most patients developed dasatinib-related chylothorax after receiving the standard dose. In this case report, we described a patient with CML who developed all 3 complications, chylothorax, pulmonary hypertension, and pericardial effusion, after receiving low-dose dasatinib for 4 years. This is rare and has not been previously reported.

## 2. Case presentation

A 43-year-old male patient was incidentally diagnosed with asymptomatic leukocytosis during a routine health examination. The patient was diagnosed with CML 6 years prior. Dasatinib (100 mg daily), initiated at diagnosis, achieved an optimal response after 1 year, with the mRNA expression level in BCR::ABL at 0.05% IS on the international scale. The patient developed bilateral pleural effusion, predominantly on the left side after 2 years of dasatinib therapy. Consequently, dasatinib was temporarily discontinued, and prednisolone was initiated. After shared decision-making between patient and the physician, dasatinib was resumed at a reduced dose of 40 mg daily. The follow-up mRNA expression level in BCR::ABL ranged from 0.0063% to 0.001% on the international scale over the next 4 years.

**Figure 1.**
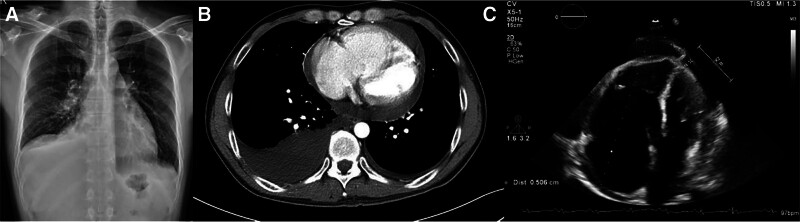
Initial imaging. (A) Chest radiography showing bilateral pleural effusion. (B) Chest computed tomography scan demonstrating bilateral pleural effusions, especially in the right lung pleural space. (C) Echocardiography illustrating a large amount of pericardial effusion.

The patient also reported progressive exertional dyspnea and an episode of near-syncope. On presentation, tachycardia (heart rate of 133 beats/min) and tachypnea (respiratory rate of 28 times/min) were observed. Chest X-ray demonstrated bilateral pleural effusions and cardiomegaly (Fig. 1A). Chest computed tomography exhibited bilateral effusions, predominantly on the right side; moderate pericardial effusion and borderline cardiomegaly; and main pulmonary artery (3.5 cm) and bilateral pulmonary artery branch dilatation, indicating pulmonary hypertension. No pulmonary mass or other parenchymal abnormalities were observed (Fig. 1B). Echocardiography was performed and revealed a large amount of pericardial effusion, a dilated right ventricle, and severe tricuspid regurgitation with an estimated pressure gradient of approximately 80 mm Hg (Fig. 1C).

Right chest thoracentesis was performed, revealing yellow milk-colored pleural fluid. The pleural fluid analysis demonstrated lymphocytic predominance (86%), lactate dehydrogenase level of 104 U/L, glucose of 106 mg/dL, protein of 5.0 g/dL, and triglycerides of 738 mg/dL. These results were consistent with a chylous effusion (Table 1). Fungal, bacterial, and tuberculosis cultures were negative. Moreover, cytopathology examination revealed no malignant cells in the pleural effusion. The patient was treated with steroids, diuretics, and sildenafil for chylothorax and pulmonary hypertension, respectively, resulting in clinical improvement and room air tolerance within 1 week. Subsequently, dasatinib was replaced with nilotinib (300 mg twice daily) for CML treatment.

**Table 1:** Pleural effusion analysis.

Variable	Result
Appearance	Milky
Color	Yellow
RBC count	3200/µL
Nucleated cell count	2371/µL
Leukocyte count	2240/µL
Neutrophil	6%
Lymphocyte	87%
Monocyte	2%
Total protein	4.2 g/dL
ADA	6 U/L
Glucose	124 mg/dL
LDH	121 U/L
Triglyceride	728 mg/dL
CEA	0.9 ng/mL

ADA = adenosine deaminase, CEA = carcinoembryonic antigen, LDH = lactate dehydrogenase, RBC = red blood cell.

**Figure 2.**
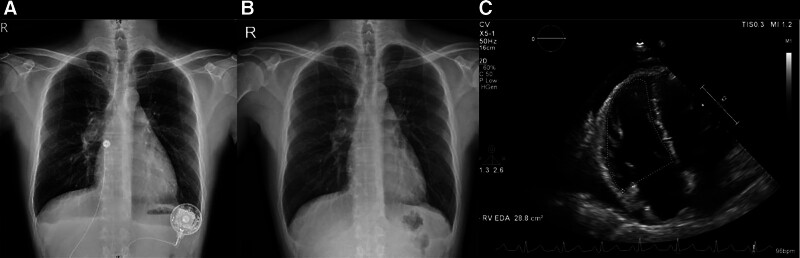
Follow-up imaging after treatment. (A and B) Chest radiography on days 5 and 28, showing bilateral pleural effusion resolution. (C) Echocardiography on day 69, demonstrating a decrease in pericardial effusion.

Follow-up chest radiography on days 5 and 28 (Fig. 2A and B) revealed bilateral pleural effusion resolution. Repeat echocardiography on day 69 (Fig. 2C) demonstrated a significant decrease in pericardial effusion volume, as well as a decreased tricuspid pressure gradient pressure.

## 3. Discussion and conclusions

Dasatinib, a second-generation TKI, is predominantly used as first- or second-line CML treatment. It inhibits BCR::ABL, the tyrosine kinase that drives CML pathogenesis.^[6,7]^ Prevalent dasatinib-related adverse effects include fluid retention, pulmonary hypertension, diarrhea, and skin rash.^[7]^ Pleural effusion is a relatively frequent adverse event, occurring in approximately 15% to 35% of patients on dasatinib therapy.^[6]^ However, chylothorax, which is a specific type of pleural effusion, is a rare dasatinib-related complication.

Chylothorax is a condition characterized by chyle accumulation in the pleural space, typically caused by the disruption or obstruction of the thoracic duct or its tributaries. This can occur due to various causes including trauma, malignancy, thoracic surgery, bacterial or tuberculosis infection, congenital anomalies, and certain medical conditions or treatments, that affect the lymphatic system.^[8,9]^ The exact mechanism and pathogenesis of dasatinib-related chylothorax remains unknown. However, Src or platelet-derived growth factor receptor beta (PDGFR-β) family kinase inhibition by dasatinib has been proposed to contribute to the pathogenesis. PDGFR inhibition interferes with the normal development and maintenance of lymphatic vessels. This disruption in lymphatic integrity may cause chyle leakage into the pleural space, resulting in chylothorax.^[10,11]^ Additionally, dasatinib is a potent inhibitor of the Src family of protein tyrosine kinases (SFKs). SFKs induce vascular endothelial growth factor expression, critical to maintaining capillary integrity.^[12,13]^ Furthermore, pericardial effusion has also been attributed to the inhibition of the PDGFR-β receptor and SFKs.^[14]^

Specifically, dasatinib is known to exert inhibitory effects on platelet aggregation, which can lead to an increased risk of bleeding in patients, though not identified in this case. This effect is primarily associated with its off-target inhibition of SFKs.^[15]^ SFKs play a critical role in platelet activation and aggregation pathways, including responses to thrombin and collagen. Even in the absence of thrombocytopenia, platelet dysfunction may contribute to bleeding events or impair vascular repair, potentially exacerbating complications like chylothorax, pleural effusion, and vascular leakage.^[16,17]^ Chylothorax and vascular complications could involve increased vascular permeability and reduced endothelial stability, both influenced by SFK inhibition. Clinicians should remain vigilant for bleeding complications, especially in patients on antiplatelet or anticoagulant therapies, and consider appropriate monitoring strategies.^[18,19]^

To date, the literature reported only a limited number of dasatinib-induced chylothorax cases. A comprehensive search of the PubMed database revealed 15 published reports.^[6–11,20–29]^ Of the 16 patients (including the present case), 14 (87.5%) were adults and 2 (12.5%) were pediatric. The gender distribution was nearly equal, with 9 males (56.3%) and 7 females (43.8%). The underlying malignancy included CML in 14 (87.5%) and Philadelphia chromosome-positive acute lymphoblastic leukemia (Ph+ ALL) in 2 (12.5%) patients. Regarding the anatomical distribution, bilateral involvement was observed in 12 (75%) patients, and most demonstrated a right-sided predominance. The time interval from dasatinib therapy initiation to chylothorax development varied considerably. In 11 patients (68.8%), chylothorax occurred within 5 years of treatment initiation. In contrast, chylothorax occurred in 5 to 10 and >10 years of dasatinib usage in 2 (12.5%) and 3 (18.8%) patients, respectively. The daily dosage of dasatinib was 100 mg in 9 patients (56.3%), 140 mg in 2 patients (12.5%), and 40 to 70 mg in 3 patients (18.8%). Concurrent complications were observed, with 4 cases (25%) of pericardial effusion and 2 cases (12.5%) of pulmonary hypertension. Our case report is the first to describe the concomitant development of both pericardial effusion and pulmonary hypertension related to dasatinib-induced chylothorax (Table 2).

**Table 2:** Reported dasatinib-induced chylothorax cases.

Age (year)	Sex	Diagnosis	Dosage (mg)	Interval from the use of dasatinib to chylothorax	Laterality	Triglyceride (mg/dL)	Pericardial effusion	Pulmonary hypertension	Treatment for chylothorax	Alternative drug	Reference
71	Male	CML	100 mg daily	6 months	Bilateral (R > L)	227	No	No	Thoracentesis, stop dasatinib, but recurrence after dasatinib resumed	N.A.	^[6]^
51	Male	CML	50 mg twice a day	50 months	Bilateral	135	N.A.	N.A.	Thoracentesis, stop dasatinib, steroid	Nilotinib	^[7]^
67	Male	CML	100 mg daily	8 months after the last dose of dasatinib	Bilateral (R > L)	969	N.A.	N.A.	Thoracentesis, diuretic, pleurodesis	N.A.	^[8]^
73	Female	CML	70 mg daily	12 months	Right	273	N.A.	N.A.	Thoracentesis, dasatinib changed to bosutinib, then imatinib; diuretic, Japanese herbal medicine “Goreisan”	Imatinib	^[9]^
72	Female	CML	100 mg daily	11 years	Right	969	No	N.A.	Thoracentesis, stop dasatinib	N.A.	^[10]^
53	Male	CML	N.A.	14 years	Left	583	N.A.	N.A.	Thoracentesis, stop dasatinib, octreotide	N.A.	^[11]^
63	Female	CML	100 mg daily	4 years	Bilateral (R > L)	700	N.A.	Right ventricle	Thoracentesis, dose reduction, then stop dasatinib	Nilotinib	^[12]^
11	Boy	CML	50 mg twice a day	33 months	Bilateral	N.A.	No	No	Thoracentesis, steroid, diuretic, octreotide, fasting, then stop dasatinib	Nilotinib	^[13]^
43	Male	CML	100 mg daily	123 months	Left	1374	N.A.	N.A.	Thoracentesis, stop dasatinib	Bosutinib	^[15]^
40	Female	CML	50 mg twice a day	40 months	Bilateral (R > L)	R: 263 L: 536	Yes	N.A.	Thoracentesis, steroid, diuretic, stop dasatinib, but recurrence after dasatinib resumed	Nilotinib	^[16]^
51	Male	Ph+ B-ALL	140 mg daily	20 months	Bilateral	R: 1346 L: 1033	N.A.	N.A.	Thoracocentesis, stop dasatinib, steroids, diuretics	Imatinib	^[17]^
39	Female	CML	50 mg daily	10 years	Bilateral	481	Yes	No	Thoracocentesis, stop dasatinib, steroids, diuretics	N.A.	^[18]^
69	Male	CML	100 mg daily	10 months	Bilateral (R > L)	405	N.A.	N.A.	Thoracentesis, dose reduction, then stop dasatinib	Bosutinib	^[19]^
5	Girl	CML	150 mg/m^2^ per day	14 months	Bilateral	603	Yes	N.A.	Thoracentesis, stop dasatinib	N.A.	^[20]^
71	Female	Ph+ ALL	140 mg daily	2 months	Bilateral	R: 625 L: 378	N.A.	N.A.	Thoracentesis, dose reduction, steroids, diuretics	N.A.	^[21]^
42	Male	CML	40 mg daily	68 months	Bilateral (R > L)	728	Yes	Yes	Thoracocentesis, stop dasatinib, steroids, diuretics	Nilotinib	Present case

CML = chronic myeloid leukemia, L = left, N.A. = not applicable, Ph+ ALL = Philadelphia chromosome-positive acute lymphocytic leukemia, R = right.

Therapeutic interventions including thoracentesis, corticosteroid administration, and diuretic therapy, together with the discontinuation of dasatinib, resulted in symptomatic improvement in all reported cases. Notably, chylothorax recurred promptly upon readministration of dasatinib in 4 patients, strongly indicating dasatinib as the causative agent. Consequently, many patients are placed on alternative CML treatment (Table 2). A comprehensive review of all available cases indicate that dasatinib-related chylothorax management includes dasatinib discontinuation, therapeutic thoracentesis to relieve respiratory compromise, diuretic therapy to help fluid removal, and a short corticosteroid course to attenuate the inflammatory response. Early recognition and prompt intervention are crucial to prevent severe complications.

Our patient developed chylothorax after 68 months of dasatinib for CML treatment. Notably, he was on a reduced dose of dasatinib for 4 years before the onset of chylothorax. Before considering dasatinib as a probable cause, other possible causes of chylothorax were excluded. Subsequently, dasatinib was discontinued and replaced with nilotinib (300 mg twice daily). Additionally, our patient was managed with methylprednisolone (40 mg daily for 4 weeks with 3 weeks of taper off), furosemide (20 mg daily for 3 days), and sildenafil (10 mg 3 times daily, which increased to 20 mg 3 times daily). Subsequent clinical follow-up revealed no chylothorax recurrence.

In conclusion, this case emphasizes the importance of recognizing the occurrence of chylothorax. This may occur despite low-dose and long-term dasatinib administration. Notably, our patient developed not only chylothorax, but also pulmonary hypertension and pericardial effusion. A combination of these 3 dasatinib-related complications in one patient has not been reported previously. In patients with CML, treated with dasatinib, chylothorax should be considered as a potential complication. To resolve the chylothorax, dasatinib discontinuation and replacement with another TKI, in addition to steroids and diuretics, are necessary. Early recognition of the condition and timely adjustment of therapy are crucial for optimizing outcomes. Close monitoring for these rare side effects is paramount even in patients on long-term or low-dose dasatinib.

## Author contributions

**Conceptualization:** Ting-Wei Pai.

**Data curation:** Cheng-Wei Huang.

**Formal analysis:** Cheng-Wei Huang.

**Investigation:** Cheng-Wei Huang.

**Methodology:** Cheng-Wei Huang.

**Project administration:** Ting-Wei Pai.

**Resources:** Cheng-Wei Huang.

**Software:** Cheng-Wei Huang.

**Supervision:** Ting-Wei Pai.

**Validation:** Cheng-Wei Huang.

**Visualization:** Ting-Wei Pai.

**Writing – original draft:** Ting-Wei Pai.

**Writing – review & editing:** Cheng-Wei Huang.
